# Chronic Otitis Media in Patients with Chronic Rhinosinusitis: A Systematic Review

**DOI:** 10.3390/medicina59010123

**Published:** 2023-01-08

**Authors:** Giuseppe Brescia, Andrea Frosolini, Leonardo Franz, Antonio Daloiso, Francesco Fantin, Andrea Lovato, Cosimo de Filippis, Gino Marioni

**Affiliations:** 1 Department of Neuroscience DNS, Otolaryngology Section, University of Padova, 35100 Padova, Italy; 2 ENT Unit, Surgical Department, Ospedali Riuniti Padova Sud, 35043 Monselice-Padova, Italy; 3 Audiology Unit, Department of Neuroscience DNS, University of Padova, 31100 Treviso, Italy; 4 Maxillofacial Surgery Unit, Department of Medical Biotechnologies, University of Siena, 53100 Siena, Italy; 5 Guided Therapeutics (GTx) International Scholarship Program, Techna Institute, University Health Network (UHN), Toronto, ON M5G2C4, Canada; 6 Otolaryngology Unit, Vicenza Hospital, 36100 Vicenza, Italy

**Keywords:** chronic otitis media, chronic rhinosinusitis, inflammation, airway, rhinosinusitis

## Abstract

*Introduction:* Chronic otitis media (COM) and chronic rhinosinusitis (CRS) are two of the most common otolaryngological disorders. CRS and COM share pathophysiological mechanisms such as bacterial infection, biofilm, and the persistence of the obstruction state of ventilation routes. The purpose of this systematic review was to evaluate all available information on the association between COM and CRS. *Methods*: The protocol of this investigation was registered on PROSPERO in November 2022. Pubmed, Scopus, Web of Science, and Cochrane databases were systematically searched according to the PRISMA statement. *Results:* After the application of inclusion-exclusion criteria, four manuscripts with adequate relevance to this topic were included in the review. The study population consisted of 20,867 patients with a diagnosis of CRS, of whom 991 were also diagnosed with COM (4.75%). *Conclusions*: The included studies have shown that CRS has become significantly associated with COMas: a global inflammatory process that involves the epithelium in both the middle ear and upper airway. The identification of a relationship between CRS and COM may contribute to preventing chronic inflammatory conditions through the early management of the associated disease. Further, carefully designed studies are necessary to demonstrate the relationship between COM and CRS.

## 1. Introduction

Chronic otitis media (COM) and chronic rhinosinusitis (CRS) are two of the most common otolaryngological disorders. However, few clinical investigations have studied the association between these two chronic inflammations.

CRS is commonly divided into two phenotype-based groups according to the presence or absence of nasal polyps; however, this classification is clearly overly simple. On the other hand, endotyping, which is based on the pathogenic mechanism, provides a precise picture that is more appropriate for use in CRS clinical practice [1]. As pathophysiological knowledge evolves, treatment protocols can be adjusted to address specific underlying disease processes [2]. COM is a heterogeneous condition defined by the persistent inflammation of the middle ear and/or mastoid cavity that can lead, in a significant number of patients, to progressive hearing loss with consequent disability and a lower quality of life [3,4,5]. COM usually develops from recurrent acute otitis media and can develop with or without cholesteatoma [6,7]. Frequent symptoms associated with the disease include otorrhea, hearing loss, and vertigo. The global prevalence of COM is estimated to be between 65 and 330 million people, and half of these individuals are projected to suffer from disabling hearing loss [8]. CRS and COM share pathophysiological mechanisms, such as bacterial infection, biofilm, and the persistence of the obstruction state of ventilation routes [9]. Otitis media occurs frequently during the evolution of CRS, even when the nasal disease is well controlled. Otolaryngologists often encounter these two diseases concurrently or note that antibiotic treatment and surgery affect one or the other’s clinical progress [10]. This finding suggests the presence, in otitis media and/or CRS, of a global inflammatory process that involves the epithelium in both the middle ear and upper airway. However, several anomalies of the nasal structures, for example, septal deviation, concha bullosa, or, generally speaking, all hypertrophic nasal pathologies, can lead to a dysfunction of the middle ear/mastoid and paranasal sinus ventilation [11] with consequent COM and CRS. Finally, it should be considered that the middle ear and paranasal sinuses have some common characteristics. First, the middle ear, paranasal sinuses, and Eustachian tubes are lined by the same pseudo-stratified columnar epithelium. Secondly, the paranasal sinuses and the middle are close and contiguous anatomic structures. Therefore, some conditions, such as gastropharyngeal reflux, viral infections, and immunologic disorders, can have inflammatory actions on both ears and paranasal sinuses [7,12,13,14,15,16,17,18].

The aim of this systematic review was to evaluate all available information on the association between COM and CRS in terms of both epidemiological and pathophysiological relationships.

## 2. Materials and Methods

### 2.1. Protocol Registration

The systematic review and meta-analysis protocol of this study was registered on PROSPERO, International prospective register of systematic reviews (Center for Reviews and Dissemination, University of York, York, UK), in November 2022 (registry number CRD42022366147).

### 2.2. Electronic Database Search

A search of the English literature published on the databases Pubmed, Scopus, Web of Science, and Cochrane was conducted according to the preferred reporting items for systematic reviews and meta-analyses (PRISMA) recommendations [19]. The search was performed from 1st January 2012 to 5th October 2022. We used the following keywords: “otitis media”; “sinusitis”. The keywords were combined accordingly on the aforementioned databases. The reference lists of all the included articles were accurately screened in order to identify other pertinent studies. The “Related articles” option present on the PubMed and Scopus homepages was also considered. The references were exported to a Zotero bibliography manager (v6.0.10, Center for History and New Media, George Mason University, Fairfax, Virginia) to remove duplicates, and then they were transposed to an Excel (Microsoft Excel 2019 for Windows 10) spreadsheet for eligibility screening.

### 2.3. Inclusion and Exclusion Criteria

**Figure 1:**
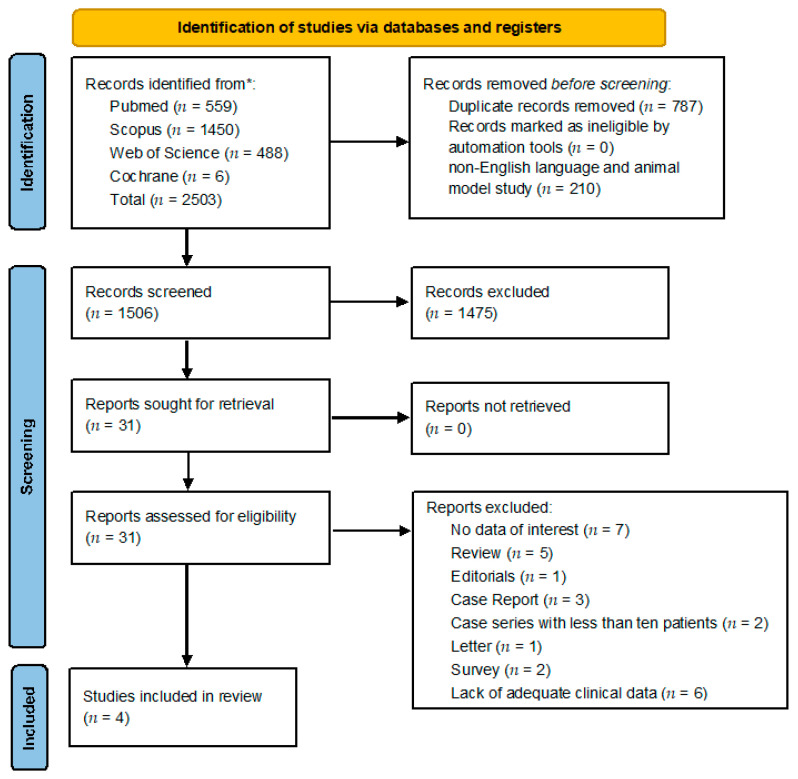
PRISMA [19] diagram resembling electronic database search and inclusion/exclusion process of the review. Legend * date of last search 5 October 2022.

An investigation was included only if the following criteria were met: (i) the inclusion of patients diagnosed with CRS who also developed COM; (ii) well-described patient evaluations (e.g., ENT diagnostic workup, radiological study, microbiological evaluation) and/or targeted treatment (e.g., medical, surgical). Exclusion criteria were: (i) articles in the form of a case report, editorial, survey, letter to the editor, or review; (ii) case series with less than 10 cases, (iii) papers with lack of adequate clinical data, (iv) animal model study and (v) non-English language (see also Figure 1).

### 2.4. Data Extraction and Quality Assessment

The authors analyzed the data from the available literature. Included studies were analyzed to extract available data and ensure the eligibility of all patients. The risk of bias was considered for all studies. Any disagreements about the inclusion/exclusion of investigations were solved by a discussion among the study team members. The quality rating of each study was categorized as poor, fair, or good, according to the National Institutes of Health quality assessment tool for Observational Cohorts and Cross-Sectional Studies [20].

## 3. Results

### 3.1. Retrieving Investigations

A total of 2503 titles were retrieved from the database search and from cross-reference checking (559 from Pubmed; 1450 from Scopus; 488 from Web of Science, and 6 from Cochrane). After the removal of duplicates, non-English language, and animal model studies, 1506 manuscripts were identified.

A selection based on title and abstract screening led to the exclusion of 1475 studies. The 31 remaining studies potentially relevant to the topic were accurately examined, and after full-text screening and the application of inclusion/exclusion criteria, four articles were included in the qualitative synthesis [10,21,22,23]. The PRISMA chart (Figure 1) summarizes the article inclusion process in this systematic review. The manuscripts retrieved and available data were insufficient to perform a quantitative synthesis of the results.

### 3.2. Quality Assessment

All the included studies had adequate relevance to the subject of this systematic review. None were randomized controlled trials; three studies were retrospective [10,21,23], whereas only one was prospective [22]. All studies were published between 2016 and 2021, and none were published in 2022.

According to the National Institutes of Health quality assessment tool for Observational Cohorts and Cross-Sectional Studies [20], only one study was rated as Good [22], one study as Fair [21], and two as Poor [10,23] because the population study consisted of heterogeneous patients who underwent medical treatment, with intranasal or oral steroids [23], or patients who had already undergone surgery (e.g., endoscopic ethmoidectomy, polypectomy, and turbinoplasty) before the study assessment [10,23]. The characteristics of the included studies are summarized in Table 1.

### 3.3. Qualitative Synthesis

#### 3.3.1. Epidemiological Characteristics

The study population consisted of 20,867 patients with a diagnosis of CRS, of which 991 were also diagnosed with COM (4.75%).

The cases analyzed by the retrieved series had an age range from 15 [23] to 85 years [10], with an overall male prevalence (of 11,791 males vs. 9076 females). A non-CRS age- and sex-matched control group was considered by only two studies [10,22], but their authors did not report clinical information about the patients. The economic conditions of the patients considered were analyzed in two studies [21,22]: Maradesha et al. [21] found an overall prevalence in low-economic-class patients, while Kuo et al. [22] described an overall prevalence in middle-class patients.

#### 3.3.2. Clinical Features

The clinical presentation consisted of CRS with or without nasal polyps (CRSsNP and CRSwNP, respectively), which was considered in all studies as inclusion criteria. Kim et al. selected the appropriate patients from the Korean Health Insurance Review and Assessment Service-National Patient Samples [10]. CRS was defined using the International Classification of Diseases (ICD-10) codes [10]. Kuo et al. [22] used the ICD-9 definitions for the diagnosis of CRS, while CRS in the Daval et al. study [23] was diagnosed according to the EPOS 2012. CRS diagnosis was confirmed by rigid nasal endoscopy in all patients in the retrieved study series. Nasal polyposis was investigated in two studies [10,23]. Kim et al. [10] found a total of 4217 CRSwNP patients and 3840 CRSsNP ones, while in the CRS population of Daval et al. [23], CRSwNP was diagnosed in 19 patients with COM and in 45 without COM. A facial CT scan was performed on the 16 patients with COM of the Daval et al. series [23]. Using the Lund-Mackay score [24], Daval et al. [23] did not find significant differences between their sub-cohorts of patients with or without COM. Maradesha et al. [21] performed temporal bone CT in 60 patients with COM without reporting significant findings. The diagnosis of COM was made after an otoscopic examination and pure-tone audiometry with tympanometry in two studies [15,17], while the other two manuscripts [10,22] did not report hearing characteristics. Daval et al. [23] reported that in their sub-cohort with COM, 16 patients had conductive hearing loss (85.0%) with a mean air-bone gap of 21.5 dB. On the other hand, Maradesha et al. [21] reported hearing loss in 93.8% of patients (76 ears out of a total of 81 ears) without specifying its degree.

The microbiological assessment was performed in only one study [21]; they collected ear discharge and the supratubal and infratubal mucopurulent or mucous discharge. Bacterial isolates from most of the patients revealed *Staphylococcus aureus*, *Pseudomonas aeruginosa*, and *Streptococcus* species from aural swabs (58.02%) and nasal swabs (60%), indicating a relevant bacteriological concordance between the ear and sinus.

## 4. Discussion

Several hypotheses have been proposed to explain the association between CRS and COM. Some research groups assumed that COM is a direct consequence of the CRS. The inflammation of the sinonasal tissue may involve the Eustachian tube determining its dysfunction, followed by the inflammation of the middle ear and mastoid [25]. On the other hand, the deviation of the nasal septum, concha bullosa, and obstructive nasal disorder pathologies can also cause a chronic ventilation alteration in the paranasal sinuses and middle ear [11].

Many previous studies have examined intimate relationships between inflammatory diseases of the ear and nose. In 1989, Finkelstein et al. [26] suggested that middle ear fluid accumulation was a relevant sign in 23% of patients with chronic sinusitis. On the other hand, abnormal radiological findings in the sinonasal district were observed in 28% of patients with otitis media, while, in the case of refractory otitis media with effusion, a frank sinusitis condition was noted in up to 78% of patients [27,28]. However, other studies were unable to show a significant linkage between COM and CRS. No correlation was found between COM and rhinosinusitis in a nationwide survey made in Korea [29].

Quite recently, the pathophysiological interpretation of CRS passed from an infectious fact to a chronic inflammatory one. CRS is thought to result from a dysfunctional immune interaction on the surface of the sinus-nasal mucosa [25]. Studies analyzing the middle ear of allergic rhinitis and asthma patients concluded that both the middle ear and nose mucosa share the same immunological features, supporting the unified airway concept [25]. Limited information is available regarding the role of other less common immunological disorders, but some findings regarding middle ear and nasal infections in primary and acquired immuno-deficiencies suggest that increased attention should be paid by the scientific community in future studies [30,31]. It was already demonstrated that COM and CRS shared the same pathogens from cultures of the middle ear and sinuses [32] and that biofilm, a cause of chronic mucosal infection, can involve both the middle ear and paranasal mucosa [33]. Regarding the possible role of recurrent and persistent viral infection in the upper airway, no results have been found in the present review. Nonetheless, it is proper to mention the recent finding of a higher HPV DNA load in COM middle ear mucosa specimens compared to normal middle ear mucosa [17] and the high prevalence of HPV infection in an analyzed group of 60 CRS patients [34].

In their 8-year observational study, Kuo et al. [22] set out to evaluate the risk of occurrence in the middle ear cholesteatoma of patients with CRS. After adjusting for potential confounders, this research group found that patients with CRS had a 69% increased risk of cholesteatoma occurrence compared with those without CRS (HR, 1.69; 95% CI, 1.23–2.32). The authors hypothesized a potential link between CRS and middle ear cholesteatoma. This research group proposed that the close monitoring of middle ear status among CRS patients might facilitate early diagnosis and prompt the treatment of comorbid cholesteatoma. Similarly, patients with cholesteatoma should be considered for CRS evaluation.

Daval et al. [23] investigated the prevalence of COM with effusion in patients with CRSwNP. This research group reported that effusive otitis media frequently occurred during the evolution of CRSwNP, even when nasal inflammation was well controlled, and they hypothesized a global inflammatory process involving the epithelium of both the middle ear and upper airways [23].

In their retrospective study, Maradesha et al. [21] investigated the role of CRS as an important COM causal factor. They emphasized the importance of etiopathology, bacteriology, and radiology for a correct diagnostic evaluation of the nose and paranasal sinuses before considering ear surgery in adults. These authors stated that bacterial CRS was the most important causative factor in the persistence of suppurative COM.

In a retrospective cohort study, Kim et al. [10] analyzed 8057 CRS and 845 COM patients to determine whether the incidence of COM in CRS patients differed from that of the control group. This research showed that CRS was significantly associated with an increased incidence of COM during a 14-year follow-up period. The incidence of COM was higher in CRS patients of all ages and genders. The authors hypothesized various mechanisms underlying this evidence. A possible cause supporting the association of these two diseases is the mechanical obstruction of the Eustachian tube. The persistent inflammatory condition caused by CRS not only thickens the mucosa of the air cells constituting the maxillary, ethmoid, and frontal sinus but also affects the mucosa of the anatomically adjacent orifice of the Eustachian tube, causing functional problems in maintaining tubal patency. Eustachian tube blockages can be aggravated by mucopurulent discharge, which may be due to a decreased mucociliary clearance and also of polypoid tissues. According to the authors, another cause for the evident association of CRS and COM could be sought in the common biomolecular mechanisms in terms of chronic inflammatory conditions. As a demonstration, the research group found an increase in cytokines in the ear and sinus mucosa [10]. Increased concentrations of interleukins (IL) such as IL-1β, IL-6, and IL-8 in the ear and sinus mucosa lead to the immune system recruitment of cells such as macrophages, dendritic cells, neutrophils, natural kills cells, and T cells. IL-6 and necrosis tumor factor-α affect the mucosa with tissue remodeling by increasing the permeability of vessels, disrupting the tight junctions between cells, and resulting in edema [10].

From a therapeutic point of view, the possible mutual pathophysiological relationship between CRS and COM may be of particular interest, especially in view of the novel advancements in non-surgical therapy. Eosinophilic CRS and COM might respond simultaneously to the biological drugs targeted against IgE and IL-5 [35]. At the same time, combined physical therapy, with nasal irrigation and Eustachian tube insufflation, might determine a clinical improvement on both the CRS and COM side [36,37]. However, further prospective clinical studies should be implemented to define what kind of treatment might provide the best clinical benefit in the subset of patients presenting with both COM and CRS.

Additionally, from a quality-of-life standpoint, data in the literature about patients with both CRS and COM are still inconclusive. In this population, the main functional concern might be hearing loss. However, the actual incidence and the social and economic burden of hearing loss in patients with CRS still need to be fully investigated.

Overall, this systematic review has some limitations: first of all, the small overall amount of studies available on this topic may be regarded as a weakness; however, being the aim of this study, the description of the specific relationship between CRS and COM, excluding other inflammatory conditions of the sinonasal district and different kinds of middle ear involvement, means that only original research papers strictly pertinent to such a topic were considered. As a result, the four considered studies included all the available literature pertinent to that topic and complied with the inclusion and exclusion criteria.

Other possible limitations include the lack of control groups in most articles and their retrospective design. However, this analysis, due to the importance and relevance of the topics covered, provides useful insights and reasons for further study. A possible study could consist of the histological analysis, also structured as recently proposed [2,38,39], to evaluate the hypothesis of the common pathogenetic mechanism of COM and CRS.

## 5. Conclusions

In conclusion, COM is a relevant condition in CRS patients. ENT specialists should pay particular attention to middle ear/mastoid examination in patients with CRS. Conversely, in the cases of COM, a rhinological evaluation is mandatory. For future directions, prospective and/or randomized studies are necessary to establish a causal relationship between these two major otolaryngology diseases. Generally speaking, COM seems more likely to be an expression of an inflammatory disease that extends to the middle ear rather than a consequence of the nasal disease itself [23]. The disclosure of a relationship between CRS and COM may contribute to preventing chronic inflammatory conditions through the early management of the associated diseases.

## Figures and Tables

**Table 1:** Summary of studies included in the review and their quality according to the National Institutes of Health quality assessment tool for Observational Cohorts and Cross-Sectional Studies [20].

Author	Year	Country	Study Type	Number of Total Patients Analyzed	Gender (M/F) No. Cases	Age	Healthy Control Group	CRS Case Group	CRS Patients Who Develop COM	Quality [14]
Maradesha et al. [21]	2016	India	Retrospective observational study	60	41/19	Mean 33 ± 11 yrs (range 18–60 yrs)	NR	60	60	Fair
Kuo et al. [22]	2017	Taiwan	Longitudinal prospective cohorts’ study	76,020	6800/5870	Mean 27.57 ± 22.03 yrs	63,350	12,670	66	Good
Daval et al. [23]	2018	France	Cross-sectional study	80	42/38	Mean 48 yrs (range 15–76 yrs)	NR	80	20	Poor
Kim et al. [10]	2021	Korea	Retrospective Cohort Study	40,285	4908/3149	40–44 yrs (747 cases) 45–49 yrs (1498 cases) 50–54 yrs (1653 cases) 55–59 yrs (1557 cases) 60–64 yrs (1180 cases) 65–69 yrs (783 cases) 70–74 yrs (425 cases) 75–79 yrs (164 cases) 80–84 yrs (43 cases) 85 + yrs (7 cases)	32,228	8057	845	Poor

Abbreviations: NR—not reported; yrs—years.

## Data Availability

The data presented in this study are available on request from the corresponding author.

## References

[1] Brescia G., Zanotti C., Parrino D., Barion U., Marioni G. (2018). Nasal polyposis pathophysiology: Endotype and phenotype open issues. Am. J. Otolaryngol..

[2] Brescia G., Alessandrini L., Marioni G. (2021). Structured histopathology for endotyping and planning rational treatment in chronic rhinosinusitis. Am. J. Otolaryngol..

[3] Aarhus L., Tambs K., Kvestad E., Engdahl B. (2015). Childhood otitis media: A cohort study with 30-year follow-up of hearing (the HUNT Study). Ear Hear..

[4] Amali A., Hosseinzadeh N., Samadi S., Nasiri S., Zebardast J. (2017). Sensorineural hearing loss in patients with chronic suppurative otitis media: Is there a significant correlation?. Electron. Physician.

[5] Elzinga H.B.E., van Oorschot H.D., Stegeman I., Smit A.L. (2021). Relation between otitis media and sensorineural hearing loss: A systematic review. BMJ Open.

[6] Chung J.H., Lee S.H., Woo S.Y., Kim S.W., Cho Y.S. (2016). Prevalence and associated factors of chronic suppurative otitis media: Data from the Korea National Health and Nutrition Examination Survey, 2009–2012: Chronic Otitis Media. Laryngoscope.

[7] Mittal R., Lisi C.V., Gerring R., Mittal J., Mathee K., Narasimhan G., Azad R.K., Yao Q., Grati M., Yan D. (2015). Current concepts in the pathogenesis and treatment of chronic suppurative otitis media. J. Med. Microbiol..

[8] Acuin J. (2007). Chronic suppurative otitis media. BMJ Clin. Evid..

[9] Marchisio P., Ghisalberti E., Fusi M., Baggi E., Ragazzi M., Dusi E. (2007). Paranasal sinuses and middle ear infections: What do they have in common?. Pediatr. Allergy Immunol..

[10] Kim S.K., Park M.W., Min C., Park I.S., Park B., Byun S.H., Choi H.G., Hong S.J. (2021). Increased risk of chronic otitis media in chronic rhinosinusitis patients: A longitudinal follow-up study using a national health screening cohort. Rhinology.

[11] Damar M., Dinc A.E., Erdem D., Biskin S., Elicora S.S., Kumbul Y. (2017). The role of the nasal and paranasal sinus pathologies on the development of chronic otitis media and its subtypes: A computed tomography Study. Niger. J. Clin. Pract..

[12] Yeo C.D., Kim J.S., Lee E.J. (2021). Association of gastroesophageal reflux disease with increased risk of chronic otitis media with effusion in adults: A nationwide population-based cohort study. Medicine.

[13] Lechien J.R., Debie G., Mahillon V., Thill M.P., Rodriguez A., Horoi M., Kampouridis S., Muls V., Saussez S. (2021). A 10-year follow-up of a randomized prospective study of 2 treatments for chronic rhinosinusitis without nasal polyps and investigation of the impact of gastroeosophageal reflux disease in the resistance to treatment. Ear Nose Throat J..

[14] Songu M., Islek A., Imre A., Aslan H., Aladag I., Pinar E., Oncel S. (2020). Risk factors for otitis media with effusion in children with adenoid hypertrophy. Acta Otorhinolaryngol. Ital..

[15] Ciprandi G., Torretta S., Marseglia G.L., Licari A., Chiappini E., Benazzo M., Tosca M.A., Marchisio P. (2020). Allergy and Otitis Media in Clinical Practice. Curr. Allergy Asthma Rep..

[16] Bemanian M.H., Rezaei K., Atighechi S., Shafiei A. (2020). The Relation of Allergy to Adenoid Hypertrophy and Otitis Media with Effusion: A Cross-sectional Study. Iran. J. Allergy Asthma Immunol..

[17] Malagutti N., Rotondo J.C., Cerritelli L., Melchiorri C., De Mattei M., Selvatici R., Oton-Gonzalez L., Stomeo F., Mazzoli M., Borin M. (2020). High Human Papillomavirus DNA loads in Inflammatory Middle Ear Diseases. Pathogens.

[18] Lam K., Schleimer R., Kern R.C. (2015). The Etiology and Pathogenesis of Chronic Rhinosinusitis: A Review of Current Hypotheses. Curr. Allergy Asthma Rep..

[19] Page M.J., McKenzie J.E., Bossuyt P.M., Boutron I., Hoffmann T.C., Mulrow C.D., Shamseer L., Tetzlaff J.M., Akl E.A., Brennan S.E. (2021). The PRISMA 2020 statement: An updated guideline for reporting systematic reviews. PLoS Med..

[20] Study Quality Assessment Tools|NHLBI, NIH. www.nhlbi.nih.gov/health-topics/study-quality-assessment-tools.

[21] Maradesha P.S., Samatha K.J., Veenapani M.K. (2016). An analysis between chronic suppurative otitis media and chronic bacterial rhinosinusitis. J. Evol. Med. Dent. Sci. JEMDS.

[22] Kuo C.L., Yen Y.C., Chang W.P., Shiao A.S. (2017). Association between middle ear cholesteatoma and chronic rhinosinusitis. JAMA Otolaryngol.-Head Neck Surg..

[23] Daval M., Picard H., Bequignon E., Bedbeder P., Coste A., Ayache D., Escabasse V. (2018). Chronic otitis media with effusion in chronic sinusitis with polyps. ENT-Ear Nose Throat J..

[24] Lund V.J., Kennedy D.W. (1997). Staging for rhinosinusitis. Otolaryngol. Neck. Surg..

[25] Hong S.N., Lee W.H., Lee S.H., Rhee C.S., Lee C.H., Kim J.W. (2017). Chronic rhinosinusitis with nasal polyps is associated with chronic otitis media in the elderly. Eur. Arch. Otorhinolaryngol..

[26] Finkelstein Y., Talmi Y.P., Rubel Y., Bar-Ziv J., Zohar Y. (1989). Otitis media with effusion as a presenting symptom of chronic sinusitis. J. Laryngol. Otol..

[27] Mills R.P., Irani B.S., Vaughan-Jones R.J., Padgham N.D. (1994). Maxillary sinusitis in children with otitis media with effusion. J. Laryngol. Otol..

[28] Hong C.K., Park D.C., Kim S.W., Cha C.I., Cha S.H., Yeo S.G. (2008). Effect of paranasal sinusitis on the development of otitis media with effusion: Influence of Eustachian tube function and adenoid immunity. Int. J. Pediatr. Otorhinolaryngol..

[29] Kim C.S., Jung H.W., Yoo K.Y. (1993). Prevalence and risk factors of chronic otitis media in Korea: Results of a nation-wide survey. Acta Otolaryngol..

[30] Yilmaz-Demirdag Y. (2011). Should newborns be screened for immunodeficiency? Lessons learned from infants with recurrent otitis media. Curr. Allergy Asthma Rep..

[31] Prasad H.K., Bhojwani K.M., Shenoy V., Prasad S.C. (2006). HIV manifestations in otolaryngology. Am. J. Otolaryngol..

[32] Brook I., Yocum P., Shah K. (2000). Aerobic and anaerobic bacteriology of concurrent chronic otitis media with effusion and chronic sinusitis in children. Arch. Otolaryngol. Neck Surg..

[33] Post J.C., Hiller N.L., Nistico L., Stoodley P., Ehrlich G.D. (2007). The role of biofilms in otolaryngologic infections: Update 2007. Curr. Opin. Otolaryngol. Head Neck Surg..

[34] Jaiswal A.S., Tanwar P., Irugu D.V.K., Sikka K., Monga R., Thakar A., Verma H. (2022). Human papilloma virus in the etiopathogenesis of allergic nasal polyposis: A prospective study. Am. J. Otolaryngol..

[35] Kagoshima H., Hori R., Kojima T., Okanoue Y., Taguchi A., Yamamoto H., Hasebe K., Shoji K. (2020). Successful treatment of eosinophilic chronic rhinosinusitis and eosinophilic otitis media using the anti-IL-5 receptor monoclonal antibody benralizumab: A case report. Respir. Med. Case Rep..

[36] Fermo S., Frosolini A., Parrino D., Chiappetta A., Marioni G., de Filippis C. (2022). Eustachian tube insufflation with thermal water: Effectiveness in the treatment of pediatric otitis media with effusion. Am. J. Otolaryngol..

[37] Franz L., Manica P., Claudatus J., Frigo A.C., Marioni G., Staffieri A. (2021). Sulfurous-arsenical-ferruginous thermal water nasal inhalation and irrigation in children with recurrent upper respiratory tract infections: Clinical outcomes and predictive factors. Am. J. Otolaryngol..

[38] Brescia G., Alessandrini L., Frasconi S., Contro G., Frigo A.C., Marioni G. (2023). Structured histopathology and laboratory evidence in nasal polyposis with different pathogenesis. Am. J. Otolaryngol..

[39] Brescia G., Alessandrini L., Giacomelli L., Parrino D., Zanotti C., Tealdo G., Franz L., Carraro V., Barion U., Marioni G. (2020). A classification of chronic rhinosinusitis with nasal polyps based on structured histopathology. Histopathology.

